# Factors associated with complementary feeding practices among children aged 6–23 months in Indonesia

**DOI:** 10.1186/s12887-022-03728-x

**Published:** 2022-12-21

**Authors:** Esti Yunitasari, Ahmad Hisyam Al Faisal, Ferry Efendi, Tiyas Kusumaningrum, Fildzah Cindra Yunita, Mei Chan Chong

**Affiliations:** 1 Faculty of Nursing, Universitas Airlangga, Surabaya, Indonesia; 2 Department of Nursing, Faculty of Medicine, University Malaya, Kuala Lumpur, Malaysia

**Keywords:** Children, Complementary feeding, Diet, Indonesia, Nutritional status

## Abstract

**Background:**

Complementary foods with breastfeeding are foods or drinks given to children aged 6–23 months to meet their nutritional needs. The non-optimal provision of complementary feeding influences malnutrition in children of this age.

**Aims:**

To analyze the factors associated with complementary feeding practices among children aged 6–23 months in Indonesia.

**Methods:**

A cross-sectional design was employed using data from the 2017 Indonesia Demographic and Health Survey. A total of 502,800 mothers with children aged 6–23 months were recruited through multistage cluster sampling. Data were analyzed using a logistic regression test to determine the correlation between predisposing, enabling, and reinforcing factors and complementary feeding practices.

**Results:**

A prevalence values of analysis showed that approximately 71.14%, 53.95%, and 28.13% of the children met MMF, MMD, and MAD, respectively. The probability of achieving minimum dietary diversity (MDD) was high in the following: children aged 18–23 months (odds ratio [OR] = 9.58; 95% confidence interval [CI] = 7.29–12.58), children of mothers with higher education (OR = 5.95; 95% CI = 2.17–16.34), children from households with upper wealth index (OR = 2.53; 95% CI = 1.85–3.48), children of mothers who received childbirth assistance by professionals (OR = 1.63; 95% CI = 1.20–2.20), and children of mothers who had access to the Internet (OR = 1.26; 95% CI = 1.06–1.50). Moreover, children from households with the upper wealth index (OR = 1.40; 95% CI = 1.03–1.91), children whose mothers were employed (OR = 1.19; 95% CI = 1.02–1.39) living in urban areas (OR = 1.28; 95% CI = 1.06–1.54) and children of mothers who received childbirth assistance by professionals (OR = 1.33; 95% CI = 0.98–1.82) were more likely to meet Minimum Meal Frequency (MMF). Finally, children aged 18–23 months (OR = 2.40; 95% CI = 1.81–3.17), of mothers with higher education (OR = 3.15; 95% CI = 0.94–10.60), from households with upper wealth index (OR = 1.41; 95% CI = 1.05–2.90) and born with professional childbirth assistance (OR = 1.82; 95% CI = 1.21–2.75) were significantly associated with minimum acceptable diet (MAD).

**Conclusions:**

The findings revealed that the prevalence of MDD and MAD in Indonesia was low. Strategies such as improving health services, economic conditions, and education level of mothers are needed to improve infant and young child feeding in Indonesia.

## Background

Stunting is a universal health problem, especially in developing countries, including Indonesia. According to the 2020 Global Nutrition Report, 149.2 million (21.9%) children aged 0–59 months experience stunting, 49.5 million (7.3%) are underweight, and 40.1 million (5.9%) are obese [1]. With over 81.7 million (54.8%) children with stunting, the Asian region is known as the home for stunted children [1]. In Indonesia, there is a high proportion of children who are stunting and have a poor nutritional status or malnutrition [1]. According to the Indonesia Basic Health Research in 2018, the proportion of children who were stuning decreased from 37.2% to 20.8% [2], and the proportion of children who were underweight and had poor nutritional status declined from 12.1% to 10.2% [2]. Despite the decreases, the prevalence of stunting and thin toddlers remains a major health problem in Indonesia [3].

Complementary foods with breastfeeding are foods or drinks given to children aged 6–23 months to meet their nutritional needs [4]. The non-optimal provision of complementary feeding influences malnutrition in children of this age; therefore, providing proper, safe, nutritious, and adequate food should be a priority during this period [5].

The World Health Organization (WHO) has defined the following indicators for infant and young child feeding (IYCF) practices: introduction of various foods from the minimum dietary diversity (MDD); minimum meal frequency (MMF); minimum acceptable diet (MAD); and solid, semi-solid, or soft foods. These indicators are used to assess IYCF practices [6–8]. In Indonesia, the prevalence of MDD among children aged 6–23 months was stagnant at 54% between 2014 to 2020, while MMF increased from 65 to 71%[9] prior to considering these indicators; nonetheless, IYCF practices remain below the recommended level.

This study aimed to determine the factors associated with complementary feeding practices (i.e., MDD, MMF, and MAD) among children aged 6–23 months in Indonesia, by considering several related factors, such as children and mother characteristics, household characteristics, access to information, and birth attendants. The findings of this study can be used as key points for identifying important interventions or policies to influence CF.

## Methods

A cross-sectional design was employed using data from the 2017 Indonesia Demographic and Health Survey.

### Population and samples

A stratified two-stage sampling technique was adopted to select the study population, which comprised households in 34 provinces in Indonesia. In the first stage, a number of census blocks with a systematic probability proportional to size and the size of households resulting from the 2010 Indonesia Public Census listing were selected. In the second stage, 25 ordinary households in each selected census block were systematically selected by updating households in each census block. The inclusion criteria were women aged 15–49 years, having the last child aged 6–23 months, and living with the child. We recruited 502,800 mothers who were eligible to participate in the study.

### Variables

The independent variables included the children’s age, which was divided into four age groups (6–8 months, 9–11 months, 12–17 months, and 18–23 months), and gender (male and female); mother’s age (15–24 years, 25–34 years, and 35–49 years), education level (uneducated, primary, secondary, and higher), and employment status (employed and unemployed); wealth index (low, lower-middle, middle, upper-middle, and upper); husband’s education level (uneducated, primary, secondary, and higher); area of residence (urban and rural); childbirth assistance (professional and non-professional); and access to the Internet (yes and no). The wealth index is defined as a composite indicator of a family’s overall standard of living, which is measured based on principal component analysis using data of household assets such as livestock, land, electronic goods, transportation equipment, and financial accounts [10].

The dependent variables were divided into three factors: MMF, MDD, and MAD. The MAD was determined based on the proportion of children who met the criteria of MDD and MMF. The MDD was defined as the consumption of four or more food groups by children on the day prior to the survey; this definition is based on the Framework of Action Complementary Feeding 2019 in Indonesia [11]. The food groups are as recommended by the WHO, consisting of dairy products, grains, roots and tubers, vegetables and fruits rich in vitamin A, other vegetables and fruits, eggs, meat, poultry, fish, shellfish, offal, and nuts. The MMF was determined by considering children who ate food according to the recommended MMF on the day prior to the survey. The MMF criteria depend on the age group of the children: two times a day for children aged 6–8 months who are breastfed, at least three times a day for children aged 9–23 months who are breastfed, and at least four times a day for children aged 6–23 months who are not breastfed [12].

### Data analysis

The data were analyzed using Stata version 16 (StataCorp LLC., Texas, USA). A chi-square test and multiple logistic regression were used to determine the relationship between the independent and dependent variables.

## Results

### Sample characteristics

Among 502,800 mothers, most (177,790 [35.36%]) had children in the 12–17 month age group and the least (79,593.2 [15.83%]) in the 6–8 month age group. According to the children’s gender, the number of male children (261,205 [51.95%]) was higher than the number of female children (241,595 [48.05%]). Regarding the mother-related variables, most mothers (52.07%) were aged 25–34 years. In terms of education level, the highest number of mothers (297,406 [59.15%]) were in the secondary (junior high school and high school) category, while the lowest was in the uneducated category (4324 [0.86%]). Most mothers (99,253 [19.74%]) came from households with an upper-middle wealth index and have status as unemployed (279,607 [55.61%]). In terms of location, most mothers (255,774 [50.87%]) lived in rural areas. During childbirth, most mothers were assisted by professionals (468,358 [93.15%]). Finally, regarding access to the Internet, just over half of the mothers (265,730 [52.85%]) had never obtained information from the Internet. The detailed information is presented in Table 1.

**Table 1:** Sociodemographic characteristics of Respondents

Variables	n	%
**Children’s age (in months)**		
6–8	79,593	15.83
9–11	87,688	17.44
12–17	177,790	35.36
18–23	157,678	31.36
**Gender**		
Male	261,205	51.95
Female	241,595	48.05
**Mother’s age (in years)**		
15–24	115,242	22.92
25–34	261,808	52.07
35–49	125,700	25
**Mother’s education**		
Uneducated	4324	0.86
Primary school	114,890	22.85
Secondary school	297,406	59.15
Higher education	86,180	17.14
**Wealth index**		
Low	98,046	19.50
Lower-middle	100,158	19.92
Middle	98,147	19.52
Upper-middle	107,197	21.32
Upper	99,253	19.74
**Mother’s occupational status**		
Unemployed	279,607	55.61
Employed	223,193	44.39
**Location**		
Urban	247,026	49.13
Rural	255,774	50.87
**Childbirth assistance**		
Professional	468,358	93.15
Non-professional	34,442	6.85
**Access to the Internet**		
Yes	237,070	47.15
No	265,730	52.85
Total	502,800	100

As per the IYCF indicators, approximately 71.14%, 53.95%, and 28.13% of the children met MMF, MMD, and MAD, respectively (Table 2).

**Table 2:** Distribution of the dependent variables based on age group

Age group	Minimum Meal Frequency (%)		Minimum Dietary Diversity (%)		Minimum Acceptable Diet (%)	
	**No**	**Yes**	**No**	**Yes**	**No**	**Yes**
6–8 months	26.39	73.61	80.57	19.43	84.68	15.32
9–11 months	31.17	68.83	53.06	46.94	74.75	25.25
12–17 months	28.80	71.20	37.45	62.55	66.21	33.79
18–23 months	28.89	71.11	34.41	65.59	70.18	29.82
Total	28.86	71.14	46.05	53.95	71.87	28.13

### Multivariate analysis

#### Minimum dietary diversity

Children aged 18–23 months [odds ratio [OR] = 9.58; 95% confidence interval [CI] = 7.29–12.58] were more likely to meet MDD than those younger, indicating that the older the child are, the greater the possibility of them meeting MDD. Meanwhile, children of mothers with higher education (OR = 5.95; 95% CI = 2.17–16.34) had a higher probability of meeting MDD compared to those of mothers with lower education levels or mothers who were uneducated. Children from households with the highest wealth index (OR = 2.53; 95% CI = 1.85–3.48] were more likely to have MDD than those from households with a lower wealth index. Moreover, children of mothers who received childbirth assistance by professionals (OR = 1.63; 95% CI = 1.20–2.20) had a higher chance of meeting MDD compared to those of mothers who were assisted by non-professionals. Finally, compared with children of mothers who never accessed the Internet at least once a month, those whose mothers did (OR = 1.26; 95% CI = 1.06–1.50) were more likely to achieve MDD (Table 3).

**Table 3:** Multivariate analysis of minimum dietary diversity

Variables	OR	*p-*value	95% CI	
			**Lower**	**Upper**
**Children’s age**				
6–8 months (*ref*)	1			
9–11 months	4.05	0.00**	3.05	5.37
12–17 months	8.23	0.00**	6.24	10.85
18–23 months	9.58	0.00**	7.29	12.58
**Mother’s education level**				
Uneducated (*ref*)	1			
Primary school	3.34	0.02**	1.24	8.97
Secondary school	3.85	0.01**	1.44	10.32
Higher education	5.95	0.00**	2.17	16.34
**Wealth index**				
Low(*ref*)	1			
Lower-middle	1.33	0.02**	1.05	1.69
Middle	1.69	0.00**	1.32	2.16
Upper-middle	1.70	0.00**	1.32	2.20
Upper	2.53	0.00**	1.85	3.48
**Mother’s occupation status**				
Unemployed (*ref*)	1			
Employee	1.00	0.95	0.85	1.1
**Location**				
Urban	1.03	0.70	0.87	1.23
Rural *(ref)*	1			
**Childbirth assistance**				
Professional	1.63	0.00**	1.20	2.20
Non-professional (*ref*)	1			
**Access to the Internet**				
Yes	1.26	0.01*	1.06	1.50
No (*ref*)	1			

^*^p < 0.5; **p < 0.05

#### Minimum meal frequency

Children aged 9–11 months [odds ratio [OR] = 0.78; 95% confidence interval [CI] = 0.60–1.01] were more likely to meet MMF than other age group of children. Children of mothers with primary school (OR = 0.56; 95% CI = 0.25–1.26) had a higher probability of meeting MMF, while children from households with the highest wealth index (OR = 1.40; 95% CI = 1.03–1.91] were more likely to have MMF than those from households with a lower wealth index. Children whose mothers were employed (OR = 1.19; 95% CI = 1.02–1.39] had a higher probability of achieving MMF than those whose mothers were unemployed. Additionally, mothers residing in urban areas (OR = 1.28; 95% CI = 1.06–1.54) and those who were born helped by a professional (OR = 1.33; 95% CI = 0.98–1.82]) were more likely to receive recommendations to meet the MFF requirements of their children than their respective counterparts (Table 4).

**Table 4:** Multivariate analysis of minimum meal frequency

Variable	OR	*p-*value	95% CI	
			**Lower**	**Upper**
**Children’s age**				
6–8 months (*ref*)	1			
9–11 months	0.78	0.06**	0.60	1.01
12–17 months	0.90	0.37*	0.71	1.13
18–23 months	0.89	0.70	0.70	1.13
**Mother’s education level**				
Uneducated	1			
Primary school	0.56	0.16*	0.25	1.26
Secondary school	0.72	0.43*	0.32	1.62
Higher education	0.74	0.48*	0.32	1.71
**Wealth index**				
Low (*ref*)	1			
Lower-middle	0.99	0.93	0.79	1.25
Middle	1.00	0.99	0.76	1.32
Upper-middle	0.89	0.40*	0.68	1.17
Upper	1.40	0.03**	1.03	1.91
**Mother’s occupation Status**				
Unemployed (*ref*)	1			
Employed	1.19	0.03**	1.02	1.39
**Location**				
Urban	1.28	0.01**	1.06	1.54
Rural *(ref)*	1			
**Childbirth assistance**				
Professional	1.33	0.07*	0.98	1.82
Non-professional (*ref*)	1			
**Access to the Internet**				
Yes	1.03	0.78	0.85	1.24
No (*ref*)	1			

^*^p < 0.5; **p < 0.05

#### Minimum acceptable diet

Children aged 18–23 months [OR = 2.40; 95% CI = 1.81–3.17) had higher odds of achieving MAD than those in the younger age groups. Similarly, children of mothers with higher education (OR = 3.15; 95% CI = 0.94–10.60) were more likely to meet MAD compared to those of mothers with lower education. Children from households with the highest wealth index (OR = 1.41; 95% CI = 1.05–2.90] were more likely to meet MAD than those from households with a lower wealth index. Furthermore, children of mothers who had received childbirth assistance by professionals (OR = 1.82; 95% CI = 1.21–2.75) were more likely to meet MAD criteria than those of mothers who were assisted by a non-professional (Table 5).

**Table 5:** Multivariate analysis of minimum acceptable diet

Variable	OR	*p-*value	95% CI	
			**Lower**	**Upper**
**Children’s age**				
6–8 months(*ref*)	1			
9–11 months	1.86	0.00**	1.39	2.50
12–17 months	2.88	0.00**	2.20	3.77
18–23 months	2.40	0.00**	1.81	3.17
**Mother’s education level**				
Uneducated	1			
Primary school	2.71	0.10*	0.82	8.96
Secondary school	2.84	0.09*	0.86	9.41
Higher education	3.15	0.06*	0.94	10.60
**Wealth index**				
Low (*ref*)	1			
Lower-middle	1.05	0.69	0.83	1.34
Middle	1.20	0.17*	0.93	1.56
Upper-middle	1.08	0.57	0.83	1.41
Upper	1.41	0.02**	1.05	2.90
**Location**				
Urban	1.05	0.62	0.87	1.25
Rural (*ref*)	1			
**Childbirth assistance**				
Professional	1.82	0.00**	1.21	2.75
Non-professional (*ref*)	1			
**Access to the Internet**				
Yes	1.04	0.67	0.87	1.25
No (*ref*)	1			

^*^p < 0.5; **p < 0.05

## Discussion

The study results indicated that infant and young child feeding practices as indicated by the CF indicators was 71.14%, 53.95%, and 28.13% for MMF, MDD, and MAD, respectively. These findings are similar to those of a previous study in which the percentage of children meeting complementary feeding among children aged 5–23 months was found to be low [13]. In the present study, sociodemographic factors influenced complementary feeding among children aged 6–23 months in Indonesia. Specifically, children aged 18–23 months of mothers with higher education, and from the highest wealth index indicated an increase in MDD and MAD. While children aged 9–11 months of mothers in the upper wealth index with status of employed and living in urban area showed significant in MMF. Similarly, children who were born with the help of a professional showed greater odds of meeting MDD, MAD and MMF. Meanwhile, children whose mothers can access internet were more likely to meet MDD.

Children aged 18–23 months showed a significant level of MDD and MAD compared to those in the younger age groups, while children aged 9–11 months were significant for MMF. From these findings, we can infer those older children are more likely to achieve MDD and MAD, on the contrary, the younger the children are, the more significant they achieve MMF. Previous studies have shown that the intention to meet MDD and MAD increases with an increase in children’s age [14–16]. This may be because, as children age and grow, they start consuming more solid foods in addition to mashed food, which increases their chance of achieving MDD and MAD at the age of 18–23 months.

Children of mothers with higher education were more likely to meet MDD and MAD compared to those of mothers with lower education. A study by Khanal et al. (2013) showed that children of mothers who had secondary (junior high and high school) and higher (university) education were more likely to meet MDD than those of mothers who did not attend school [7]. These results are consistent with those of Joshi et al. (2012) [17] and Sekartaji et al. (2021) [18]. A study by Kambale et al. (2021) found that children whose mothers had secondary and higher education were more likely to meet MAD [19]. Similarly, Sebayang et al. (2020) revealed that higher knowledge levels in women were associated with meeting children’s dietary needs [4]. This may be because mothers with higher education have better knowledge regarding children’s health and nutritional programs [20–22]. Moreover, mothers with formal education are likely to ensure feeding their children based on the nutritionist’s advice and in accordance with their child’s age. Considering that the 6–23 month period is critical for children’s growth, complementary feeding must be optimized to support their development and prevent malnutrition [23].

In this study, children belonging to families with a higher wealth index had a higher probability of meeting MMD, MAD and MMF than those belonging to families with a lower wealth index. A study by Dhami et al. (2019) in India showed that households with the highest wealth index were significantly related to the fulfilment of MDD [24]. Similarly, a study by Nkoka et al. (2018) in Malawi found that children of mothers with middle, upper-middle, and upper wealth indexes were more likely to meet MDD and MAD [6]. These results are consistent with those of Dhami et al. (2019)’s study in India, where households in the top wealth index met MDD requirements of the children [24]. These trends may have been observed because mothers with the highest wealth index are easily able to obtain various healthy and nutritious foods for their children.

Children of working mothers had a higher likelihood of achieving MMF compared to those of mothers who were unemployed. Working mothers have a greater intention to meet MMF requirements of their children than those who do not work [25, 26]. The present study suggests that workplace environments should uphold women’s rights by ensuring that female workers with children aged 6–23 months have the space and time to breastfeed their children. In addition, families need to be involved in the complementary feeding practice while the mothers are at work, thus promoting this practice among working mothers.

Children living in urban areas had a high probability of meeting MMF. A study conducted in Ethiopia found that mothers who lived in urban areas were more likely to meet the MMF criteria for their children than those who lived in rural area [27]. This may be a result of mothers having more access to diverse foods or information about complementary feeding, which could increase the likelihood of them providing healthy and nutritious foods to their children as recommended by nutritionists or health practitioners.

Mothers who had received childbirth assistance by professionals were more likely to provide MDD, MAD and MMF. Previous studies by Sema et al. (2021), Dhami et al. (2019), and Temesgen et al. (2018) showed that mothers who gave birth in health facilities had a higher chance of meeting MDD than those who gave birth at home [24, 28, 29]. Professional birth attendants provide opportunities to mother to obtain postnatal care information, especially information on feeding guidelines for children to provide balanced nutrition. Thus, mothers are likely to be exposed to useful information about complementary feeding.

Children of mothers who had access to the Internet at least once a week were more likely to meet MDD. This result is in line with research conducted by Beyene et al. (2015) in Northwest Ethiopia [8] and Nkoka et al. (2018) in Malawi [6], which showed that mothers who were exposed to mass media were more likely to meet the MDD requirements of their children than those who did not. Mass media, such as the Internet, provide nutritional health information from credible and trusted sources; moreover, exposure to different media platforms allows mothers to update their knowledge on the nutritional requirements of children and increase their awareness of others. The concept of utilizing mass media for health education has been implemented in Malawi, where information on maternal health services, including antenatal and postnatal care [30], was provided. Our study suggests that mass media should be increasingly used as a tool to spread nutrition awareness and knowledge in order to encourage the behavioral change in providing breastfeeding among mothers.

Regarding to our statistical analysis, we found that MMF and MMD are high but MAD is too low number. This data is resulted as the current phenomenon in Indonesia: even though MMF has been met the standard, but this does not reflect that MMD meet the standard. Thus, the samples that meet MMF and MMD recorded as the low number.

### Implications for practice

The present study suggests that for improving the prevalence of MDD, MFF, and MAD, health practitioners should provide adequate nutritional information and nutritional education to mothers. Aside from improving the economic condition of families and education level of parents, information about complementary feeding practices needs to be provided to the public, either by using mass media or enhancing health services at the primary care level. In addition, intersectoral collaboration to address nutritional problems at the community level should be promoted and incorporated with government interventions.

### Limitation

This was a cross-sectional study so causality between the variables cannot be determined.

## Conclusions

The prevalence rates of MDD and MAD are relatively low in Indonesia. Children’s age, wealth index, mother’s education level, and childbirth assistance were associated with MDD and MAD. Meanwhile, the mother’s occupation and area of residence were related to MMF. Improving health services, economic status, and education level of mothers is essential for boosting IYCF in Indonesia.

## Data Availability

The data used in this study are available online from the 2017 IDHS website under the “Individual Recode” section. Access to the dataset requires registration and is granted only for legitimate research purposes. A guide for applying for dataset access is available at https://dhsprogram.com/data/Access-Instructions.cfm.

## Abbreviations

CI: Confidence interval
IYCF: Infant and young child feeding
MAD: Minimum acceptable diet
MDD: Minimum dietary diversity
MMF: Minimum meal frequency
OR: Odds ratio
WHO: World Health Organization
